# Frequency, Prognosis, and Clinical Features of Unexpected versus Expected Cardiac Arrest in the Emergency Department: A Retrospective Analysis

**DOI:** 10.3390/jcm13092509

**Published:** 2024-04-24

**Authors:** Karolina Szaruta-Raflesz, Tomasz Łopaciński, Mariusz Siemiński

**Affiliations:** Department of Emergency Medicine, Medical University of Gdańsk, M. Skłodowskiej-Curie 3a Street, 80-210 Gdańsk, Poland; karolina.szaruta-raflesz@gumed.edu.pl (K.S.-R.); tomasz.lopacinski@gumed.edu.pl (T.Ł.)

**Keywords:** emergency department, unexpected cardiac arrest, in-hospital cardiac arrest

## Abstract

**Background**: Though out-of-hospital CA (OHCA) is widely reported, data on in-hospital CA (IHCA) and especially cardiac arrest (CA) in the emergency department (CAED) are scarce. This study aimed to determine the frequency, prevalence, and clinical features of unexpected CAED and compare the data with those of expected CAED. **Methods**: We defined unexpected CAED as CA occurring in patients in non-critical ED-care areas; classified as not requiring strict monitoring. This classification was the modified Japanese Triage and Acuity Scale and physician assessment. A retrospective analysis of cases from 2016 to 2018 was performed, in comparison to other patients experiencing CAED. **Results**: The 38 cases of unexpected CA in this study constituted 34.5% of CA diagnosed in the ED and 8.4% of all CA treated in the ED. This population did not differ significantly from other CAED regarding demographics, comorbidities, and survival rates. The commonest symptoms were dyspnoea, disorders of consciousness, generalised weakness, and chest pain. The commonest causes of death were acute myocardial infarction, malignant neoplasms with metastases, septic shock, pulmonary embolism, and heart failure. **Conclusions**: Unexpected CAED represents a group of potentially avoidable CA and deaths. These patients should be analysed, and ED management should include measures aimed at reducing their incidence.

## 1. Introduction

Cardiac arrest (CA) in the emergency department (CAED) can be viewed as a subtype of in-hospital CA (IHCA). Though out-of-hospital CA (OHCA) is widely reported, data on IHCA and especially CAED are scarce. According to Polish data, IHCA occurs in 0.3% of hospitalised patients, and CAED accounts for 6.8% of IHCA patients [1]. According to data from the American National Registry of Cardiopulmonary Resuscitation, CAED constitutes 12.1% of total IHCA cases [2]. The most available data indicate that patients experiencing CAED have better prognoses in terms of survival and neurological outcomes than patients experiencing CA in other hospital departments [3,4]. However, there are also reports that survival could be higher among CA in other hospital departments than CAED, but it could be associated with a higher rate of known causes of cardiac arrest in this group than in emergency department patients. Many a time, these patients are also diagnosed and observed for a long time in order tro assess what could help better predict the risk of cardiac arrest [5].

It is hypothesised that better prognosis of CAED patients is the result of ED (Emergency Department) staff being better prepared to undertake resuscitation [3,6]. There is a certain population in whom CA is highly probable, for example, patients who experience OHCA or patients who have undergone severe trauma. Such patients remain under continuous observation and intensive monitoring to allow immediate notification of CA and prioritised commencement of resuscitation. However, there is another population of CAED patients initially classified as those who are not at risk of CA and who do not require monitoring. As those patients are not strictly monitored and CA in those cases is unexpected for ED staff, we have termed the condition as ‘unexpected CAED’ (unex-CAED) [7].

To the best of our knowledge, such a population has not yet been clearly defined and described yet. Available data pay attention to the increased frequency of myocardial ischaemia, arrhythmia, respiratory insufficiency, and sepsis in this group of patients [5,8]. Intuitively, we believe that improvement in the identification of such subjects will allow avoidance of CA, and fatal cases of unex-CAED should be regarded as potentially avoidable deaths.

As no data on such a population of patients are available, we aimed to analyse the following aspects: to create an easy-to-use and universal definition of unex-CAED, to assess the frequency of unex-CAED, to seek repetitive clinical features of patients undergoing unex-CAED, and to compare the clinical features and prognosis of patients with unexpected CAED with those having expected CAED.

## 2. Materials and Methods

### 2.1. Study Design and Settings

This retrospective study was performed between 1 January 2016 and 31 December 2018 in a 1100-bed university hospital with a trauma centre. It is located within a three-city agglomerated region, inhabited by approximately 1,000,000 citizens. There are 120,000 hospitalisations annually, with 30,000–33,000 ED visits per year. The ED of the hospital is one of the four EDs serving the population of agglomeration. 

There are four physicians and nine nurses/paramedics constantly on duty. The ED is divided into the following areas:

- A waiting area dedicated for patients classified as stable, who do not require any monitoring or acute therapy or who are waiting for their visit, results, or discharge;
- An observation area dedicated to patients classified as stable, requiring acute therapy or a low to moderate level of monitoring. This is also an area for patients waiting for boarding to other units (20 beds);
- A shock room dedicated to patients requiring immediate intensive therapy/resuscitation (e.g., victims of polytrauma or CA) (2 beds);
- An intensive-therapy room dedicated to patients requiring intensive monitoring and therapy because of their general instability (4 beds).

All patients visiting the ED undergo triage, which is based upon the modified Japanese Triage and Acuity Scale (mJTAS) [9]. The triage nurse, after measuring the vital signs of a patient and obtaining a short medical history, labels the patient with one of the following triage colours:

- Red (1) for patients requiring immediate resuscitation;
- Orange (2) for patients in the ED;
- Yellow (3) for patients in need of urgent visits;
- Green (4) for patients in need of a visit of low urgency;
- Blue (5) for patients without urgent conditions.

Unstable haemodynamic patients are always referred for monitoring. We have also detailed the rules for the procedure of re-triage. Patients should be re-assessed after the triage assessment if it is not possible for the doctor examine him in a timely manner: the “orange patient” every 5 min, the “yellow” every 20 min, the “green” every 60 min and the “blue” every 90 min. Most of the patients we analyzed had already undergone examination by a doctor and were not subject to the procedure of re-triage. 

### 2.2. Definition of Unexpected Cardiac Arrest in the Emergency Department

For this study, we used a useful technical definition of unexpected CA. We aimed to find all cases in which CA occurs in a subject who is classified as ‘stable’, not requiring intense monitoring. All patients classified as threatened by CA are allocated to the intensive therapy area or shock room (resuscitation area). Therefore, we defined unex-CAED as CA in patients classified as not requiring intense monitoring and who remained in the waiting area or observation room (areas that are not designated for patients who have an immediately life-threatening condition). 

### 2.3. Selection of Participants

Patients experiencing CA were identified by unique ICD-10 codes (I46.0, I46.1, and I46.9). Demographic data of the patients, results of triage assessment, data on the course of CA and resuscitation, further history of the patients, and data on comorbidities were extracted from the hospital information system. Patients who were admitted to the ED after OHCA were excluded from this study, even if CA reappeared during their stay in the ED. As the in-hospital protocol of resuscitation requires precise descriptions of the context of observed CAs, analysis of patients’ charts allowed the division of CAED into two groups: expected CAED (observed in the shock room or the intensive care area) and unexpected CAED (observed in all other areas of the ED). All CAED were included in this study, without any further exclusion or inclusion criteria.

### 2.4. Outcome Measures

The primary outcome measures of this study were the frequency and survival rate of unex-CAED and the main symptoms of patients who experienced unex-CAED. The secondary outcome measures were the demographic features and comorbidities of patients who experienced unex-CAED and the final diagnoses of patients who experienced unex-CAED. 

### 2.5. Analysis

An anonymising software (MedStreamDesigner, Transition Technologies, Warsaw, Poland) was used to extract requisite data from the hospital information system. The extracted data were subjected to statistical analyses. Descriptive analyses were performed, and expected CAED and unex-CAED were compared using the Student’s *t*-test for continuous values and the chi-square test for discrete values. Continuous values were presented as means with standard deviations, and discrete values were presented as numbers with percentages. A two-tailed *p*-value of <0.05 was considered statistically significant. 

### 2.6. Ethics Approval and Consent to Participate

The study protocol was approved by the local ethical committee of the author’s institute: Independent Bioethical Committee for Scientific Studies at the Medical University of Gdansk (Approval number NKBBN/140/2021). As this study was based upon a retrospective analysis of anonymized medical charts, no written consent was obtained from the patients, which was described in the application for ethical approval which was accepted by committee. The Independent Bioethical Committee for Scientific Studies at the Medical University of Gdansk waived informed consent from all the patients for this study. All methods were carried out in accordance with the Declaration of Helsinki.

## 3. Results

### 3.1. Characteristics of the Study Participants

**Figure 1:**
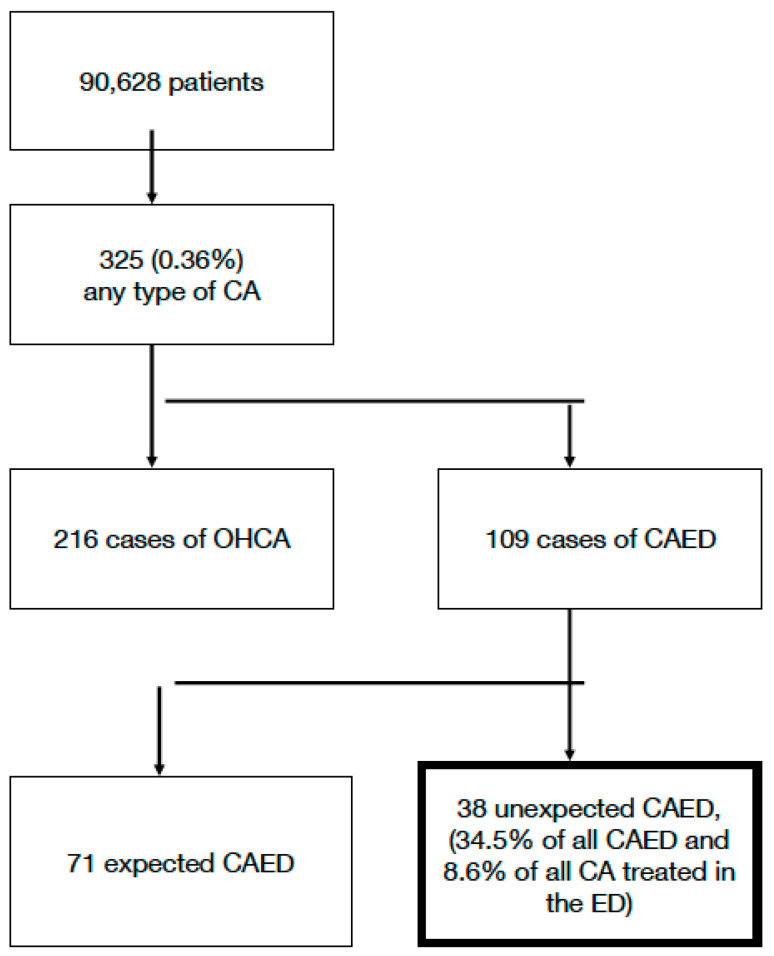
Flowchart of patients.

In total, 90,628 patients were admitted to the ED during the study period, with 325 cases of any type of CA (0.36%) treated in the ED. We found 216 cases of OHCA and 109 cases of CAED. Among these, 38 cases were classified as unex-CAED, constituting 34.5% of all CAED and 8.6% of all CA cases treated in the ED. The flowchart of patients is presented in Figure 1.

The demographic data of patients with CAED are presented in Table 1. There were no significant differences in age, sex, or comorbidities between patients who experienced expected CAED and unex-CAED. 

### 3.2. Main Results

Data on the clinical features of CA and its final outcome are presented in Table 2. There were no statistically significant differences between the expected and unexpected CAED groups in terms of electroencephalographic recording, number of returns of spontaneous circulation (ROSC), or the final functional outcome. The only significant difference was noticed in cardiac rhythms that were observed during CA, with pulseless electrical activity (PEA) being more prevalent in unexpected CA and asystole occurring more frequently in expected CA.

The commonest clinical symptom in the unex-CAED group was dyspnoea, followed by reduced consciousness and generalised weakness. The commonest cause of CA was coronary syndrome, followed by dissemination of malignant disease and septic shock. Three unex-CAED (7.9%) cases occurred before triage and another three (7.9%) before physician examination. Eighteen (47.4%) cases occurred in patients who were classified as requiring hospitalisation in a regular ward and waiting for allocation to such a ward. The triage results, main symptoms, and final diagnoses (verified during autopsy) of unexpected CAED are presented in Table 3. 

## 4. Discussion

To the best of our knowledge, the present study is the first to describe a new subtype of CAED: CA that occurs in patients who are initially assessed as not requiring immediate therapy or strict monitoring. Generally, data on CAED are scarce; moreover, we have not come across reports of studies that analysed similarly specified subpopulations: patients who underwent CA were classified as stable. We believe that this population is important and requires further attention. It may be hypothesised that proper classification of these subjects could lead to the prevention of CA in some cases. Therefore, at least some deaths resulting from unex-CAED could be classified as potentially avoidable deaths.

Avoidable deaths and avoidable CA represent a significant part of in-hospital CA and in-hospital deaths. Hodgetts found that 62% of primary in-hospital CA (including those of the ED) were potentially avoidable. Moreover, the risk of primary CA increased in patients staying in a non-critical area, and 17% of CA occurred in patients hospitalised in an area not appropriate to their conditions. In total, 95% of the latter CA cases were classified as potentially avoidable, which corresponds well with our definition of unex-CAED [10]. Rogne et al., in a recent study, found a lower frequency of avoidable in-hospital deaths (6.7%) but still suggested the importance of this issue [11]. Therefore, clinical characterisation of patients with unex-CAED may lead to improvements in patient safety. 

The frequency of CA described in our study was comparable to that in other studies focusing on CAED [12,13]. We did not find data on unex-CAED from other centres for comparison. Our analysis showed that patients with unex-CAED did not differ from patients with expected CA in terms of age, sex, and known comorbidities. In both groups, unshockable rhythms were more frequent, which is in agreement with data from other studies [14]. Successful ROSC and survival until the end of hospitalisation were more frequent in unex-CA patients, although the difference did not reach statistical significance. This trend can be explained by the fact that those subjects were initially in a better clinical condition (none of them received the highest triage priority). It remains consistent with the observation that CAED have better prognoses [3].

Our data show certain options for avoiding ex-CAED. We found three cases of unexpected CAED that occurred directly after the arrival of patients in the ED (all three patients who arrived the ED by ambulance). The initial assessment of patients in the pre-hospital phase could suggest a risk for CA. Therefore, pre-notification in such cases would allow the ED to be prepared for patients in need of urgent intensive therapy [15,16]. Our data suggest that dyspnoea, disorders of consciousness, generalised unspecified weakness, and chest pain are the commonest symptoms in patients experiencing unexpected CA. Therefore, patients with these symptoms should be monitored immediately after arrival at the ED. A noticeable fraction of unexpected CA occurred in patients diagnosed and waiting for boarding to other wards. Their presence in the ED is a consequence of ED overcrowding, which has been described as a factor that increases the risk of CAED patients [13,17]. The ED, which plays a crucial role in any healthcare system, requires many measurements of quality of care. The measures proposed thus far include the length of stay, boarding time, and number of patients who leave without being seen by a doctor [18,19]. The frequency of unex-CAED may successfully serve as a measurement index of the efficiency of the triage system and the medical examination for determining which patients are at risk of sudden CA.

By definition, the unex-CAED created unexpected outcomes in patients admitted to the ED. Our data showed that there was no difference in the efficacy of resuscitation between patients experiencing unex-CAED and those experiencing ex-CAED. Nevertheless, the predictability of unexpected CA is a good index of staff readiness in undertaking resuscitation. Therefore, we postulate that the effectiveness of resuscitation in unexpected CAED may serve as a routine measure of how well team members are prepared for starting resuscitation. 

We use mJTAS at our department. We are aware that studies comparing various triage systems are scarce; therefore, it is difficult to implement our results to an environment with another triage system, e.g., MTS. As our main conclusion is that triage assessments are definitely not sufficient for the prognostication of sudden cardiac arrest at the ED, and a similar analysis should be performed in departments using other triage systems. 

The most important criterion upon which the decision of intensive supervision of the patient was made was the priority (colour) assigned during triage. Therefore, all patients with highest priority (red), which indicates suspected hemodynamic instability and features of shock, were allocated to the shock room or intensive therapy area of the ED. According to our definition, those cases were not classified as unexpected cardiac arrest, and they are not described in this analysis. The strongest conclusion from our observation is that in some cases triage colour-based allocation is not sufficient.

We would like to provoke with our paper a discussion on unexpected cardiac arrest at emergency department. The cases of unex-CAED described in this study happened regardless of triage, physician assessment, and observation. Therefore, there must be some causes of such events beyond the obvious functions of the ED. The first step towards such a discussion should be precise monitoring of all cardiac arrests and deaths in the ED with analyses on whether they were expected or not.

### Limitations

Our study has some limitations. The sample size analysed in the present study was small. Observations from a longer timespan or multicentre studies focusing on unex-CAED should facilitate the collection of more information. Another limitation of this project is that the small number of patients described does not allow for generalisation of the findings to different populations. Studies with such a protocol are at risk of selection bias; however, we formulated the inclusion/exclusion criteria using a general and obvious allocation method to ensure that the sample selection was not biased. As our study was observational, we could not prove the causality of unex-CAED. Our preliminary results should be verified in prospective, multicentre studies with larger samples of patients.

## 5. Conclusions

In summary, our data show that there is a population of ED patients who, despite having a low triage score and undergoing an initial physician’s assessment, remain at a high risk of unexpected CA. Therefore, each ED should analyse the frequency of such episodes and implement mechanisms to prevent unex-CAED. Future research should focus on the epidemiology of unex-CAED, with studies based upon nationwide or global registers. Another interesting direction of investigation is the prophylaxis of unex-CAED, for example, through precise analysis of the triage results of participants who experience unex-CAED.

## Figures and Tables

**Table 1:** Demographic features and comorbidities of patients who experienced expected and unexpected cardiac arrest in the emergency department. CA: Cardiac arrest; CAED: Cardiac arrest in the emergency department; OHCA: Out-of-hospital cardiac arrest.

	Unex-CAED (*n* = 38)	CAED (*n* = 71)	*p*
Sex (M/F) (*n*/*n*; %/%)	20/18; 52.6%/47.4%	35/36; 49.3%/50.7%	0.8413
Age, years, (mean ± SD)	71.5 ± 15.6	72.7 ± 12.4	0.7186
Comorbidities, *n* (%):			
Cardiac insufficiency	11 (28.9%)	15 (21.1%)	0.4797
Chronic kidney disease	9 (23.7%)	10 (14.1%)	0.2890
Neoplastic disease	10 (26.3%)	16 (22.5%)	0.6465
Coronary disease	6 (15.8%)	14 (19.7%)	0.7960
Dementia	1 (2.6%)	4 (5.6%)	0.6561
Alcohol dependency	1 (2.6%)	7 (9.9%)	0.2571
Pathologic obesity	1 (2.6%)	4 (5.6%)	0.6561
Hypertension	17 (44.7%)	28 (39.4%)	0.0001
Atrial fibrillation	7 (18.4%)	11 (15.5%)	0.0537
Cardiomyopathy	1 (2.63%)	3 (4.2%)	1.0000
COPD ^1^	2 (5.3%)	9 (12.7%)	0.3229
Chronic anaemia	0 (0.0%)	2 (2.8%)	0.5416
Hepatic cirrhosis	0 (0.0%)	5 (7.0%)	0.1606

^1^ COPD, chronic obstructive pulmonary disease.

**Table 2:** Data on cardiac arrest, resuscitation, and survival for expected and unexpected cardiac arrests.

	Unex-CAED (*n* = 38)	CAED (*n* = 71)	*p*
Successful ROSC, *n* (%)	17 (44.7%)	24 (33.8%)	0.3025
Deaths at ED, *n* (%)	21 (55.3%)	47 (66.2%)	0.3025
Death during hospitalisation, *n* (%)	5 (13.2%)	11 (15.5%)	1
Dependent at exception, *n* (%)	10 (26.3%)	20 (28.2%)	1
Time to resuscitation, minutes (mean ± SD)	12.6 ± 20.2	14.6 ± 17.9	0.4084
First observed cardiac rhythm			
VF, *n* (%)	4 (10.5%)	3 (4.2%)	0.2351
VT, *n* (%)	1 (2.6%)	3 (4.2%)	1
PEA, *n* (%)	24 (63.2%)	28 (39.4%)	0.0265
Asystole, *n* (%)	7(18.4%)	35 (49.3%)	0.0019
Unknown, *n* (%)	2 (5.3%)	2 (2.8%)	0.6096
Defibrillation during CPR, *n* (%)	5 (13.1%)	6 (8.4%)	0.5103
Total number of deaths, *n* (%)	26 (68.4%)	58 (81.2%)	0.1519

CA: cardiac arrest; ED: emergency department; SD: standard deviation; CPR: cardiopulmonary resuscitation; ROSC: return of spontaneous circulation; VF: ventricular fibrillation; VT: ventricular tachycardia; PEA: pulseless electrical activity; SD: standard deviation; ns: not significant.

**Table 3:** Triage results, main symptoms, and final diagnoses in the unexpected CAED group.

	Unex-CAED (*n* = 38)	CAED Group (*n* = 71)
**Triage results (colour given)**		
Red	0 (0.0%)	40 (56.3%)
Orange	13 (34.2%)	29 (40.8%)
Yellow	15 (39.5%)	2 (2.8%)
Green	7 (18.4%)	0 (0.0%)
Blue	0 (0.0%)	0 (0.0%)
CA before triage	3 (7.9%)	0 (0.0%)
**Main symptoms**		
Dyspnoea	15 (39.5%)	34 (47.9%)
Disturbances of consciousness	9 (23.7%)	32 (45.1%)
Weakness	8 (21.1%)	22 (31.1%)
Chest pain	7 (18.4%)	12 (16.9%)
Abdominal pain	6 (15.8%)	6 (8.5%)
Lower limb pain	4 (10.5%)	0 (0.0%)
Vomiting	5 (13.2%)	6 (8.5%)
Fever	4 (10.5%)	3 (4.2%)
Head injury	3 (7.9%)	2 (2.8%)
Politrauma	0 (0.0%)	1 (1.4%)
Seizures	0 (0.0%)	1 (1.4%)
**Final diagnoses**	**(13 cases were verified during autopsy)**	**(9 cases were verified during autopsy)**
Acute myocardial infarction	7 (18.4%)	9 (12.7%)
Malignant neoplasms with metastases	5 (13.2%)	19 (26.8%)
Septic shock	7 (18.4%)	10 (14.1%)
Pulmonary embolism	4 (10.5%)	5 (7.0%)
Heart failure	4 (10.5%)	5 (7.0%)
Pneumonia	3 (7.9%)	0 (0.0%)
Ileus	2 (5.3%)	0 (0.0%)
Rupture of aortic aneurysm	1 (2.6%)	5 (7.0%)
Oesophageal varices bleeding	1 (2.6%)	4 (5.6%)
Cardiac tamponade	1 (2.6%)	0 (0.0%)
Takotsubo cardiomyopathy	1 (2.6%)	0 (0.0%)
Alcohol intoxication	1 (2.6%)	1 (1.4%)
Haemopneumothorax	1 (2.6%)	0 (0.0%)
Intracranial haemmorhage	0 (0.0%)	2 (2.8%)
Politrauma	0 (0.0%)	1 (1.4%)
Aspiration	0 (0.0%)	2 (2.8%)
Chronic renal disease	0 (0.0%)	1 (1.4%)
Senility	0 (0.0%)	7 (9.9%)

## Data Availability

Data is available at the Corresponindg Author on request.
